# Perioperative magnetic resonance imaging in breast cancer care: Distinct adoption trajectories among physician patient-sharing networks

**DOI:** 10.1371/journal.pone.0265188

**Published:** 2022-03-15

**Authors:** Xiao Xu, Pamela R. Soulos, Jeph Herrin, Shi-Yi Wang, Craig Evan Pollack, Brigid K. Killelea, Howard P. Forman, Cary P. Gross

**Affiliations:** 1 Department of Obstetrics, Gynecology and Reproductive Sciences, Yale School of Medicine, New Haven, Connecticut, United States of America; 2 Cancer Outcomes, Public Policy and Effectiveness Research Center, Yale School of Medicine, New Haven, Connecticut, United States of America; 3 Department of Internal Medicine, Yale School of Medicine, New Haven, Connecticut, United States of America; 4 Department of Chronic Disease Epidemiology, Yale School of Public Health, New Haven, Connecticut, United States of America; 5 Department of Health Policy and Management, Johns Hopkins University Bloomberg School of Public Health, Baltimore, Maryland, United States of America; 6 Department of Medicine, Johns Hopkins University School of Medicine, Baltimore, Maryland, United States of America; 7 Johns Hopkins University School of Nursing, Baltimore, Maryland, United States of America; 8 Hartford HealthCare Medical Group, Bridgeport, Connecticut, United States of America; 9 Department of Radiology and Biomedical Imaging, Yale School of Medicine, New Haven, Connecticut, United States of America; Dartmouth College Geisel School of Medicine, UNITED STATES

## Abstract

**Background:**

Despite no proven benefit in clinical outcomes, perioperative magnetic resonance imaging (MRI) was rapidly adopted into breast cancer care in the 2000’s, offering a prime opportunity for assessing factors influencing overutilization of unproven technology.

**Objectives:**

To examine variation among physician patient-sharing networks in their trajectory of adopting perioperative MRI for breast cancer surgery and compare the characteristics of patients, providers, and mastectomy use in physician networks that had different adoption trajectories.

**Methods and findings:**

Using the Surveillance, Epidemiology, and End Results-Medicare database in 2004–2009, we identified 147 physician patient-sharing networks (caring for 26,886 patients with stage I-III breast cancer). After adjusting for patient clinical risk factors, we calculated risk-adjusted rate of perioperative MRI use for each physician network in 2004–2005, 2006–2007, and 2008–2009, respectively. Based on the risk-adjusted rate, we identified three distinct trajectories of adopting perioperative MRI among physician networks: 1) low adoption (risk-adjusted rate of perioperative MRI increased from 2.8% in 2004–2005 to 14.8% in 2008–2009), 2) medium adoption (8.8% to 45.1%), and 3) high adoption (33.0% to 71.7%). Physician networks in the higher adoption trajectory tended to have a larger proportion of cancer specialists, more patients with high income, and fewer patients who were Black. After adjusting for patients’ clinical risk factors, the proportion of patients undergoing mastectomy decreased from 41.1% in 2004–2005 to 38.5% in 2008–2009 among those in physician networks with low MRI adoption, but increased from 27.0% to 31.4% among those in physician networks with high MRI adoption (p = 0.03 for the interaction term between trajectory group and time).

**Conclusions:**

Physician patient-sharing networks varied in their trajectory of adopting perioperative MRI. These distinct trajectories were associated with the composition of patients and providers in the networks, and had important implications for patterns of mastectomy use.

## Introduction

Perioperative magnetic resonance imaging (MRI) is increasingly used in breast cancer surgery [1]. However, despite its high sensitivity which helps define the extent of disease and detect multifocal/multicentric/contralateral diseases, perioperative MRI can produce false positives and may overestimate the extent of disease [2–5]. Its identification of additional cancers and overestimation of tumor size can lead to the use of more extensive surgery, i.e., mastectomy as opposed to breast conserving surgery. Perioperative MRI may also result in indeterminate findings, causing concerns by patients and their request for mastectomy [6]. Empirical evidence has shown that perioperative use of breast MRI increases patients’ likelihood of undergoing mastectomy [7] yet there is no benefit of perioperative MRI in improving patients’ short- and long-term outcomes such as risk of re-operation, cancer recurrence, and survival [3, 5, 8–11]. This raises concerns about overutilization of perioperative MRI and a need to understand factors that led to the rapid adoption.

The social contagion theory posits that physicians’ clinical decisions are influenced by the attitudes and practice of other physicians via personal or professional interactions [12–14]. Recent research on physician patient-sharing networks–defined as groups of physicians who frequently share patients with each other [15]–showed that use of perioperative breast MRI varied from 0% to 81% across physician patient-sharing networks [16]. Moreover, surgeons were more likely to adopt perioperative breast MRI when they frequently shared patients with early adopters [16]. This suggests an important role of physician peer influence in the uptake of breast MRI.

Time to adoption of new technology often exhibits an S-shaped curve, where the rate of adoption is typically slow in the initial phase where there are few adopters and in the late phase where there are few nonadopters [17, 18]. The period in-between, however, is usually when most of the adoptions occur and where there can be large variability in rate of adoption. This provides a critical window for studying overutilization of unproven technology such as perioperative breast MRI. A positive deviance approach can be particularly helpful in this setting for understanding how and why some physician patient-sharing networks avoid overutilization and maintain slower adoption of perioperative MRI [19].

Although perioperative breast MRI was introduced into clinical practice in the 1990’s, its utilization increased most rapidly in the second half of the 2000’s [1, 20, 21]. Therefore, we used data from 2004–2009 to examine how physician patient-sharing networks differed in their trajectories of adopting perioperative breast MRI and the characteristics of physician networks that had low versus high rates of adoption. To inform downstream consequences of overutilizing perioperative MRI, we further examined the association between a physician network’s MRI adoption trajectory and their patients’ risk of undergoing mastectomy (as opposed to breast conserving surgery). Findings from this study may inform strategies for addressing overutilization of other unproven or low-value technologies.

## Materials and methods

### Study overview

Using the linked Surveillance, Epidemiology, and End Results (SEER)‐Medicare database, we identified physician patient-sharing networks and estimated risk-adjusted rates of perioperative breast MRI use for each physician network in 2004–2005, 2006–2007, and 2008–2009; we purposely selected these time-periods, during which adoption of perioperative MRI accelerated most rapidly in clinical practice [1, 20, 21]. Based on these risk-adjusted rates of MRI use, we applied a novel growth mixture modeling technique to identify latent classes of physician patient-sharing networks that had different trajectories of adopting perioperative MRI. We compared physician network characteristics across the distinct adoption trajectories. We also examined temporal changes in the utilization of mastectomy versus breast conserving surgery to evaluate potential consequences of the distinct MRI adoption trajectories.

### Data and sample

**Fig 1:**
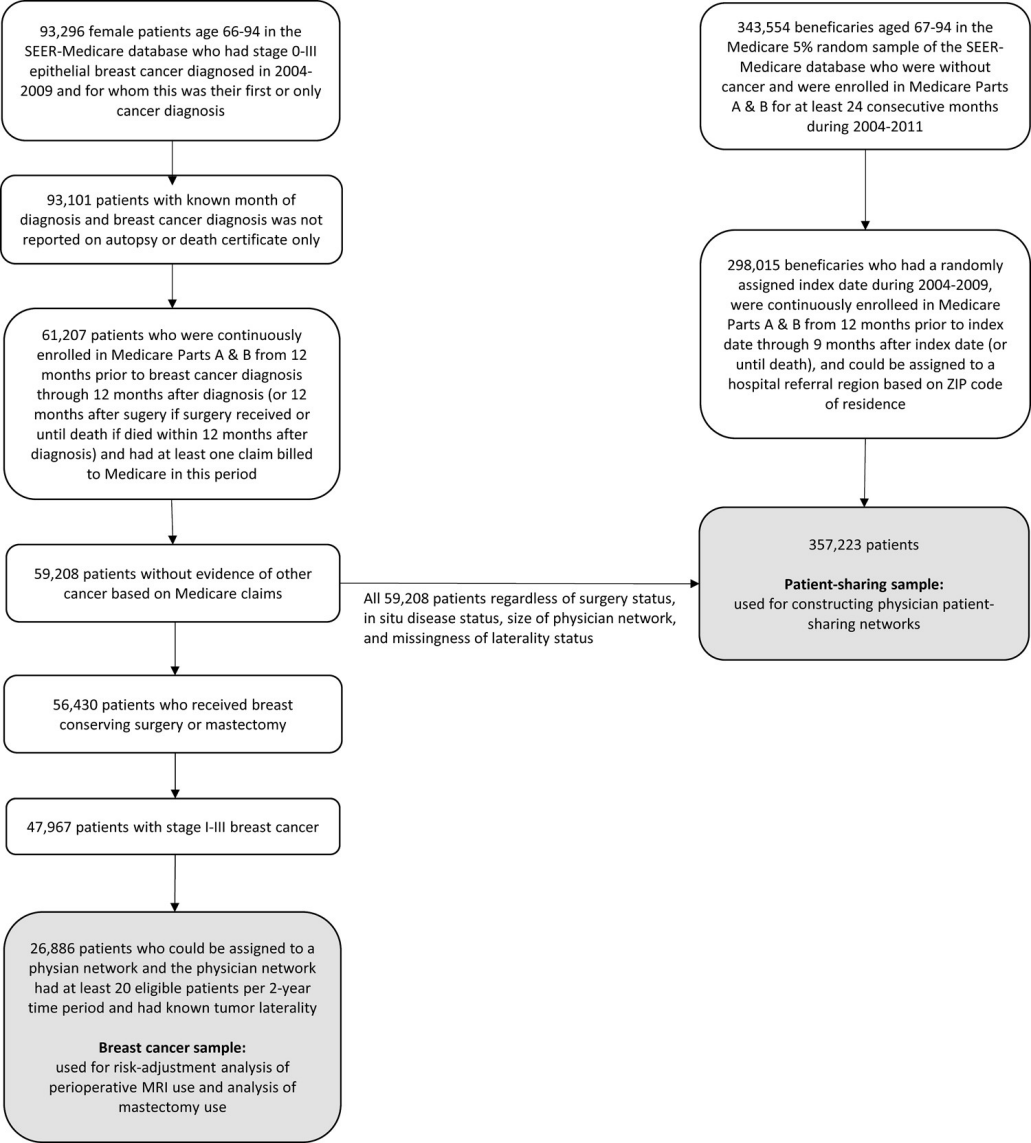
Sample selection criteria. SEER = Surveillance, Epidemiology, and End Results.

Data for this study came from the Surveillance, Epidemiology, and End Results (SEER)-Medicare database [22]. SEER-Medicare contains detailed tumor characteristics of patients with cancer along with their linked Medicare claims, as well as a random 5% sample of Medicare beneficiaries who reside in geographic areas of the SEER registries but do not have cancer [22]. We used data from 2004–2009 corresponding to the time-period during which use of perioperative MRI accelerated most rapidly making it an ideal time window for studying physicians’ adoption behavior. The Yale University Human Investigation Committee Biomedical Institutional Reviewer Board determined that this study did not constitute human subjects research since the study only involved secondary analysis of de-identified data. Sample selection criteria are detailed below, as well as in Fig 1.

#### Breast cancer sample

Our analysis of MRI use included women 66–94 years of age who had a primary diagnosis of stage I-III epithelial breast cancer with known laterality and underwent a surgery. We excluded patients if: 1) the breast cancer was not their first cancer diagnosis, 2) their breast cancer was of non‐epithelial histology, 3) their month of breast cancer diagnosis was unknown, 4) their diagnosis of breast cancer was reported only on autopsy/death certificate, 5) they were not continuously enrolled in Medicare parts A and B for 12 months before diagnosis through 12 months after surgery (or until death if died within 12 months after surgery), 6) they had no claims billed to Medicare in the 12 months before through 12 months after diagnosis, 7) they had another cancer diagnosed between the diagnosis of breast cancer through 12 months following breast cancer surgery, and 8) their breast cancer laterality was unknown. The requirement about continuous enrollment in Medicare parts A and B before and after diagnosis enabled accurate measurement of a patient’s medical history and therapies received. We used two-year intervals to define time-periods (2004–2005, 2006–2007, and 2008–2009). In order to have a reasonable sample size (i.e., number of eligible patients with breast cancer) within each physician network to allow for accurate estimation of its rate of perioperative MRI use, we restricted the breast cancer sample to patients in physician patient-sharing networks with ≥20 eligible patients in each time-period. To facilitate linkage to physician patient-sharing networks, patients must also have a mailing ZIP code that could be assigned to a hospital referral region (HRR) [23].

#### Patient-sharing sample

Construction of physician patient-sharing networks also used the SEER-Medicare database but included a broader sample of patients to enhance robustness of the networks. This broader sample included: 1) women in the breast cancer sample described above, 2) women meeting all other eligibility criteria for the breast cancer sample described above regardless of the size of their physician network, 3) women meeting all other eligibility criteria for the breast cancer sample described above but who had in situ disease, did not receive surgery, or had unknown laterality, and 4) non-cancer patients identified using the Medicare 5% random sample in the SEER-Medicare database. Each non-cancer patient was assigned a random index date and met the same age and Medicare enrollment criteria as the breast cancer sample.

### Physician patient-sharing networks

Physician patient-sharing networks are clusters of physicians who frequently share patients with each other [15]. When constructing physician patient-sharing networks, we included surgeons, as well as physicians in several other specialties who commonly provide care for patients with breast cancer—medical oncologists, radiation oncologists, radiologists, and primary care physicians. Primary care physicians were identified based on specialty information in Medicare carrier claims and included physicians in the following specialties: general practice, family practice, internal medicine, obstetrics/gynecology, geriatric medicine, and gynecological oncology. Inclusion of non-surgeons helped enhance robustness of the physician patient-sharing networks constructed. Unique physicians were determined using the encrypted National Provider Identifier on Medicare claims. To enhance reliability of the physician patient-sharing networks constructed and focus on physicians who had a reasonable patient volume, we required that a physician need to have at least five eligible patients in order to be included in the construction of physician patient-sharing networks [24].

Patient-sharing relationships were identified using Medicare claims from the patient-sharing sample (see prior section) billed in the 3 months before through 9 months after patients’ diagnosis/index date. Physicians who billed for the same patient were considered to “share” the patient. To ensure measurement of meaningful connections between physicians, two physicians needed to share at least two patients in order to be considered linked [24]. Physicians were assigned to HRRs based on their patients’ HRR (physicians who treated patients from multiple HRRs were assigned to more than one HRR and their data in different HRRs were analyzed separately) [24]. Following the Girvan-Newman algorithm, we identified patient-sharing networks by dividing physicians in each HRR into mutually exclusive networks and progressively removing connections between physicians that were farthest from other physicians in the network until goodness of fit was optimized [24–26]. The identified physician patient-sharing networks are hereinafter referred to as physician networks for short.

Cancer patients were assigned to physician networks based on their primary surgeon. For each cancer patient, we identified her primary surgeon based on the National Provider Identifier associated with her surgery claims. If multiple surgeons were listed, we used the Current Procedural Terminology (CPT)/Healthcare Common Procedure Coding System (HCPCS) modifier code to distinguish primary vs. assistant surgeon. When needed, we also gave precedence to surgeons with a specialty in general surgery or surgical oncology.

### Measures

For each patient in the breast cancer sample, we measured her use of perioperative MRI (yes/no). Perioperative MRI was defined as an MRI received 3 months before diagnosis through 3 months following surgery. This helped capture preoperative MRI for surgical planning, as well as postoperative MRI for assessing residual disease to guide the performance of additional surgery [27, 28]. MRI use was identified based on relevant CPT/HCPCS codes (see S1 Table).

For each patient in the breast cancer sample, we also measured the type of surgery she received (mastectomy versus breast conserving surgery). This was determined by searching Medicare claims in the 9 months following the diagnosis of breast cancer for relevant CPT and International Classification of Diseases 9th revision (ICD-9) procedure codes (see S1 Table). Patients who received both types of surgery within the 9-month window were considered to have had mastectomy.

To account for the impact of potential confounding factors, we measured patients’ age, tumor characteristics (cancer stage, grade, tumor size, lymph node involvement, hormone receptor status, laterality), and comorbidities. Comorbidities were measured using the Elixhauser index based on ICD-9 diagnosis codes [29, 30]. Patient sociodemographic characteristics were also available to help characterize the sample, including race, marital status, residence in a metropolitan area, and census tract/ZIP code-based median household income.

For each physician patient-sharing network, we measured its characteristics based on its patient composition and physician composition. Measures of patient composition included number of breast cancer patients (i.e., those meeting eligibility criteria for the breast cancer sample) in the network, mean income among these patients (based on census tract/ZIP code-specific median household income), and the proportion of these patients that were Black, resided in a metropolitan area, or had a visit with their primary care physician in the past year. These variables helped inform potential differences in patient socioeconomic status and access to care across physician networks. For physician composition, we measured the number of physicians in each network and the percentage of these physicians that were medical oncologists, radiation oncologists, radiologists, surgeons, and primary care physicians.

### Statistical analysis

A patient’s likelihood of undergoing perioperative MRI (yes/no) was estimated using logistic regression with a generalized estimating equation approach (to account for clustering of patients within physician network). The regression adjusted for patients’ age, cancer stage, grade, tumor size, lymph node involvement, hormone receptor status, laterality, number of comorbidities, and the time-period when cancer was diagnosed (2004–2005, 2006–2007, or 2008–2009). After estimating this regression, we calculated each patient’s expected probability of undergoing perioperative MRI based on coefficient estimates from the model and conditioning on the patient’s clinical risk factors and the time-period when her cancer was diagnosed. Then for each physician network in a given time-period, we divided the sum of observed perioperative MRI use among all its patients in this time-period by the sum of expected perioperative MRI use among these patients, multiplying by the sample overall observed rate of perioperative MRI use in the corresponding time-period.

Using these time-period specific, risk-adjusted rates of MRI use, we applied a growth mixture modeling approach to identify latent classes of physician networks that exhibited distinct trajectories of adopting perioperative MRI [31]. Physician networks within the same latent class shared a similar trajectory, whereas physician networks in different latent classes had distinct trajectories. We compared alternative models with an increasing number of trajectories and selected the optimal number of trajectories based on the Bayesian information criterion [31]. The trajectories were modeled as a linear function of time because empirical evidence suggested no significant quadratic term of time. Each physician patient-sharing network was assigned to a trajectory based on its maximum posterior probability of “membership” (i.e., probability that the physician network belonged to a given trajectory given its observed data) [31].

Since the identified trajectories exhibited low to high levels of MRI adoption, we used the Kendall’s tau-b correlation coefficient to examine the association between physician network characteristics (e.g., proportion of a network’s physicians being a surgeon) and the rank order of the trajectory that a network belonged to. A sensitivity analysis comparing physician network characteristics across the distinct trajectories using Kruskal-Wallis test (i.e., without accounting for the ordinal nature of the trajectories) produced similar results.

To inform potential mechanisms of the differential adoption of perioperative MRI across the distinct trajectories, we also calculated the following metrics for each surgeon: 1) whether any of his/her patients used perioperative MRI (yes/no) in each time-period (i.e., any use), 2) if the surgeon did not use perioperative MRI at baseline (2004–2005), what proportion of his/her patients used perioperative MRI in later time-period (i.e., intensity of use among early non-users), and 3) if the surgeon used perioperative MRI at baseline (2004–2005), what proportion of his/her patients used perioperative MRI in each time-period (i.e., intensity of use among early users). Then we summarized and compared these metrics for surgeons across the distinct trajectories.

To compare differences in use of mastectomy among patients in physician networks that had the distinct trajectories of MRI adoption, we estimated a logistic regression for a patient’s likelihood of undergoing mastectomy (versus breast conserving surgery) as a function of indicators for the distinct MRI trajectories, time-period, and their interaction. The interaction terms informed whether the distinct MRI adoption trajectories might affect temporal trends in mastectomy use. The regression adjusted for patients’ age, cancer stage, grade, tumor size, lymph node involvement, hormone receptor status, laterality, and number of comorbidities. A generalized estimating equation approach was used to account for clustering of patients within physician networks.

P values <0.05 were considered statistically significant. Analyses were conducted using R 3.5.1 and igraph 1.0 (R Foundation for Statistical Computing, Vienna, Austria), SAS 9.4 (SAS Institute, Cary, NC), and Stata 14.2 (StataCorp LP, College Station, TX).

## Results

### Sample characteristics

A total of 26,886 patients from 147 physician patient-sharing networks met eligibility criteria for the breast cancer sample. Patients’ mean age was 75.8 years (standard deviation = 6.7). Most patients (58.3%) had stage I disease, while 32.0% and 9.7% had stage II and III disease, respectively (Table 1). Among the 147 physician networks, the median (interquartile range [IQR]) volume was 145 (99–226) patients and 133 (90–204) physicians (Table 2). The number of physician networks per HRR ranged from 1 to 9, and 97 of the 147 physician networks were in HRRs that had more than one physician network.

**Table 1:** Characteristics of patients in the breast cancer sample (N = 26,886 patients).

Characteristics	Overall	Perioperative MRI		
	N (%)	Yes	No	P Value
		N (%)	N (%)	
Sample size	26,886	5,302	21,584	
Age				<0.001
66–69	5,839 (21.7)	1,702 (32.1)	4,137 (19.2)	
70–74	6,688 (24.9)	1,603 (30.2)	5,085 (23.6)	
75–79	6,344 (23.6)	1,132 (21.4)	5,212 (24.1)	
80–84	4,878 (18.1)	620 (11.7)	4,258 (19.7)	
85–94	3,317 (11.7)	245 (4.6)	2,892 (13.4)	
Race				<0.001
White	24,229 (90.1)	4,890 (92.2)	19,339 (89.6)	
Black	1,725 (6.4)	227 (4.3)	1,498 (6.9)	
Other	932 (3.5)	185 (3.5)	747 (3.5)	
Marital status				<0.001
Married	1,1894 (44.2)	2,817 (53.1)	9,077 (42.1)	
Unmarried	13,929 (51.8)	2,249 (42.4)	11,680 (54.1)	
Unknown	1,063 (4.0)	236 (4.5)	827 (3.8)	
Residence in a metropolitan area				<0.001
Yes	23,760 (88.4)	4,868 (91.8)	18,892 (87.5)	
No	3,126 (11.6)	434 (8.2)	2,692 (12.5)	
Area-level median household income^a^				<0.001
<$33K	4,649 (17.3)	608 (11.5)	4,041 (18.2)	
$33K-$40K	3,889 (14.5)	577 (10.9)	3,312 (15.3)	
$40K-$50K	5,955 (22.1)	1,075 (20.3)	4,880 (22.6)	
$50K-$63K	5,685 (21.1)	1,190 (22.4)	4,495 (20.8)	
≥$63K	6,708 (24.9)	1,850 (34.9)	4,856 (22.5)	
Number of comorbidities				<0.001
0	14,737 (54.8)	3,356 (63.3)	11,381 (52.7)	
1–2	9,516 (35.4)	1,654 (31.2)	7,862 (36.4)	
≥3	2,633 (9.8)	292 (5.5)	2,341 (10.8)	
Stage				
I	15,682 (58.3)	3,138 (59.2)	12,544 (58.1)	0.02
II	8,603 (32.0)	1,703 (32.1)	6,900 (32.0)	
III	2,601 (9.7)	461 (8.7)	2,140 (9.9)	
Grade				<0.001
1	6,825 (25.4)	1,411 (26.6)	5,414 (25.1)	
2	11,801 (43.9)	2,440 (46.0)	9,361 (43.4)	
3	6,808 (25.3)	1,188 (22.4)	5,620 (26.0)	
4	217 (0.8)	27 (0.5)	190 (0.9)	
Missing	1,235 (4.6)	236 (4.5)	999 (4.6)	
Tumor size, cm				<0.001
<2	17,066 (63.5)	3,523 (66.4)	13,543 (62.7)	
2–5	8,454 (31.4)	1,512 (28.5)	6,942 (32.2)	
>5	1,183 (4.4)	225 (4.2)	958 (4.4)	
Missing	183 (0.7)	42 (0.8)	141 (0.7)	
Node positive				0.42
No	20,404 (75.9)	4,001 (75.5)	16,403 (76.0)	
Yes	6,482 (24.1)	1,301 (24.5)	5,181 (24.0)	
Hormone receptor status				<0.001
Negative	3,654 (13.6)	670 (12.6)	2,984 (13.8)	
Positive	21,878 (81.4)	4,450 (83.9)	17,428 (80.7)	
Unknown	1,354 (5.0)	182 (3.4)	1,172 (5.4)	
Tumor laterality				0.19
Right-sided	13,234 (49.2)	2,653 (50.0)	10,581 (49.0)	
Left-sided	13,652 (50.8)	2,649 (50.0)	11,003 (51.0)	

MRI = magnetic resonance imaging.

^a^ Fewer than 11 patients with unknown area level median household income were included in the middle category ($40K-$50K) due to privacy concerns.

**Table 2:** Characteristics of physician patient-sharing networks, overall and across the distinct trajectory groups (N = 147 physician patient-sharing networks).

Network Characteristics	Overall	Distinct Adoption Trajectory			
		Low Adoption	Medium Adoption	High Adoption	Kendall’s Tau-b Rank Correlation
	(N = 147)	(N = 85)	(N = 48)	(N = 14)	
Patient composition					
Number of breast cancer patients	145 (99, 226)	119 (94, 216)	156.5 (110.5, 270)	137.5 (93, 180)	0.09 (p = 0.16)
Area-level median household income	49,972 (41,644, 60,254)	46,733 (39,197, 57,138)	54,519 (45,813, 66,168)	59,287 (49,972, 69,767)	**0.23 (p<0.001)**
Proportion of breast cancer patients who were Black (%)	2.6 (0.7, 7.0)	4.4 (0.9, 8.7)	1.8 (0.7, 5.0)	1.3 (0.0, 3.8)	**-0.17 (p = 0.009)**
Proportion of breast cancer patients residing in a metropolitan area (%)	98.9 (82.5, 100.0)	98.3 (81.7, 100.0)	99.7 (82.3, 100.0)	100.0 (95.2, 100.0)	0.09 (p = 0.21)
Proportion of breast cancer patients who had a PCP visit in the past year (%)	93.8 (92.2, 95.3)	93.6 (92.2, 95.1)	93.9 (91.7, 95.2)	95.2 (93.3, 96.2)	0.07 (p = 0.31)
Physician composition					
Number of physicians	133 (90, 204)	116 (87, 200)	142 (103, 228)	133 (82, 183)	0.04 (p = 0.51)
Proportion of physicians who were					
Surgeons (%)	10.7 (9.1, 12.4)	11.0 (9.2, 12.4)	10.5 (9.1, 12.4)	10.0 (8.2, 12.2)	-0.06 (p = 0.40)
Medical oncologists (%)	7.1 (5.7, 8.8)	6.7 (5.3, 8.5)	7.8 (6.3, 9.2)	8.2 (6.2, 12.8)	**0.18 (p = 0.008)**
Radiation oncologists (%)	4.3 (3.1, 6.3)	4.1 (2.9, 6.1)	4.1 (3.1, 5.9)	7.2 (5.7, 9.9)	**0.15 (p = 0.02)**
Radiologists (%)	23.2 (18.6, 27.8)	22.8 (18.2, 26.3)	24.1 (20.2, 28.3)	24.8 (18.0, 31.6)	0.09 (p = 0.16)
Primary care physicians (%)	53.6 (47.3, 59.0)	55.5 (48.0, 59.5)	52.7 (46.9, 57.7)	48.2 (43.6, 54.4)	**-0.14 (p = 0.03)**

PCP = primary care physician.

Data reported as median (interquartile range). Bold font indicates correlation coefficients that are statistically significant.

### Overall use of perioperative MRI

Overall, 5,302 patients (19.7%) received perioperative MRI during our study period, increasing from 7.7% in 2004–2005 to 30.9% in 2008–2009. Among these patients, 4,984 (94.0%) received the MRI preoperatively, while the remaining 318 (6.0%) received it postoperatively. Compared to patients who did not undergo perioperative MRI, those who received perioperative MRI were younger, had fewer comorbidities, were more likely to be white and married, and were more likely to reside in a metropolitan area or an area with higher household income (p<0.001 for all) (Table 1). However, differences in their tumor characteristics were modest (Table 1).

### Distinct trajectories of adopting perioperative MRI

The 147 physician networks varied substantially in MRI use even after adjusting for patient tumor characteristics and other clinical risk factors. The median (IQR) risk-adjusted rate of perioperative MRI was 4.4% (0–11.1%) in 2004–2005 and 25.1% (11.9–43.0%) in 2008–2009.

**Fig 2:**
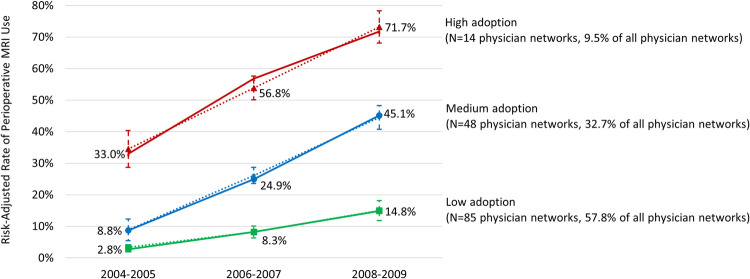
Distinct trajectories of adopting perioperative MRI among physician patient-sharing networks. MRI = magnetic resonance imaging. Rate of perioperative MRI use reflects risk-adjusted rate for each physician patient-sharing network in each time-period after accounting for differences in patient tumor characteristics and other clinical risk factors. Solid lines reflect mean risk-adjusted rate among physician patient-sharing networks in each trajectory. Dotted lines reflect trajectories predicted by the growth mixture model, with error bars reflecting 95% confidence intervals.

The growth mixture model identified three distinct trajectories of adoption among the 147 physician networks: 1) low adoption (risk-adjusted rate of MRI use increased from 2.8% in 2004–2005 to 14.8% in 2008–2009), 2) medium adoption (8.8% to 45.1%), and 3) high adoption (33.0% to 71.7%) (Fig 2). These trajectories accounted for 57.8%, 32.7% and 9.5% of the physician networks, respectively, and 56.4%, 35.6%, and 8.0% of the patients, respectively.

Physician networks that had a larger proportion of medical oncologists (Kendall’s tau-b correlation = 0.18, p = 0.008) or radiation oncologists (Kendall’s tau-b correlation = 0.15, p = 0.02) were more likely to follow a trajectory with higher MRI adoption (Table 2). In contrast, physician networks that had a larger proportion of primary care physicians were less likely to follow a trajectory with higher MRI adoption (Kendall’s tau-b correlation = -0.14, p = 0.03).

Physician networks that had higher mean income among patients were more likely to have a trajectory with higher MRI adoption (Kendall’s tau-b correlation = 0.23, p<0.001) (Table 2). Yet physician networks that cared for a larger proportion of Black patients tended to follow a trajectory with lower MRI adoption (Kendall’s tau-b correlation = -0.17, p = 0.009).

Table 3 summarized changes in the proportion of surgeons using perioperative MRI and the intensity of their utilization over time across the three trajectories. Compared to surgeons in the low adoption trajectory, those in the high adoption trajectory had a more rapid increase in rate of utilization among early non-users who adopted perioperative MRI, as well as further elevated utilization among early users.

**Table 3:** Changes in the proportion of surgeons using perioperative MRI and intensity of utilization over time across the distinct trajectories (N = 1,969 surgeons).

Time-Period	Low Adoption (N = 1,163)	Medium Adoption (N = 670)	High Adoption (N = 136)
Proportion with any use: Whether a surgeon had any patients using perioperative MRI			
2004–2005	11.9%	30.5%	58.8%
2006–2007	25.1%	49.0%	74.0%
2008–2009	39.2%	69.2%	83.5%
Increase between 2004–2005 and 2008–2009	27.3 percentage points	38.7 percentage points	24.7 percentage points
Mean intensity of use among early non-users: What proportion of a surgeon’s patients used perioperative MRI (if the surgeon did not use perioperative MRI at baseline)			
2004–2005	0%	0%	0%
2006–2007	5.7%	13.8%	38.4%
2008–2009	10.2%	34.3%	55.8%
Increase between 2004–2005 and 2008–2009	10.2 percentage points	34.3 percentage points	55.8 percentage points
Mean intensity of use among early users: What proportion of a surgeon’s patients used perioperative MRI (if the surgeon used perioperative MRI at baseline)			
2004–2005	22.8%	27.1%	46.1%
2006–2007	19.9%	32.8%	52.0%
2008–2009	23.0%	47.1%	69.7%
Increase between 2004–2005 and 2008–2009	0.2 percentage points	20.0 percentage points	23.6 percentage points

MRI = magnetic resonance imaging.

### Association with surgical management

**Fig 3:**
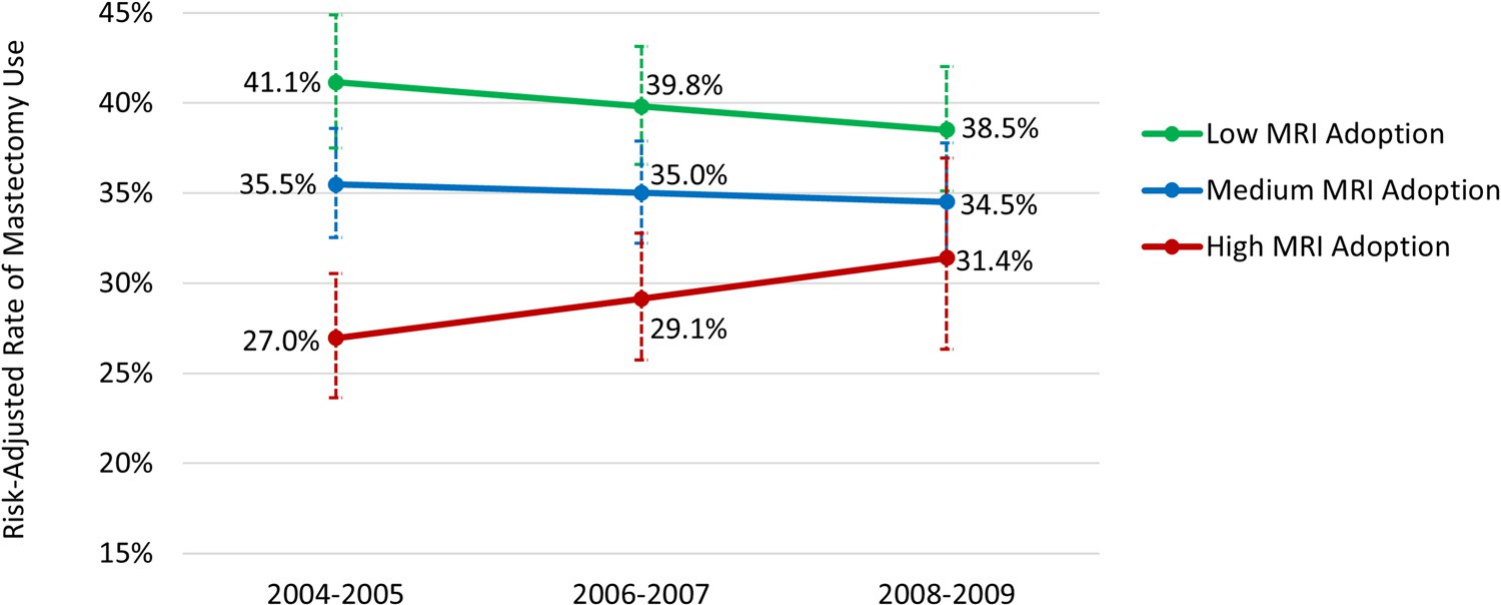
Risk-adjusted proportion of patients using mastectomy over time, by the distinct trajectories of MRI adoption. MRI = magnetic resonance imaging. Error bars reflect 95% confidence intervals.

Based on adjusted results from multivariable regression analysis and sample mean patient characteristics, Fig 3 shows the different patterns of mastectomy use over time in physician networks that had the distinct trajectories of MRI adoption. The proportion of patients undergoing mastectomy decreased from 41.1% in 2004–2005 to 38.5% in 2008–2009 among patients in physician networks that had a trajectory of low MRI adoption, but increased from 27.0% in 2004–2005 to 31.4% in 2008–2009 among patients in physician networks that had a trajectory of high MRI adoption (p = 0.03 for the interaction term between trajectory group and time). Use of mastectomy was relatively stable among patients in physician networks that had medium MRI adoption (35.5% in 2004–2005 versus 34.5% in 2008–2009).

## Discussion

We found large variation among physician patient-sharing networks in their trajectory of adopting perioperative MRI during a time-period when it was rapidly adopted into breast cancer care nationwide. Physician networks in the higher adoption trajectory tended to have a larger proportion of cancer specialists, more patients with high income, and fewer patients who were Black. Physician networks with low MRI adoption experienced a reduction in mastectomy use.

A unique contribution of this study is the distinct “phenotypes” of physician networks identified with regard to their trajectories of adopting perioperative MRI. While use of perioperative MRI reached 71.7% in some physician networks in 2008–2009, it remained much lower at 14.8% in other physician networks. Understanding the characteristics of these physician network “phenotypes” may inform opportunities to decelerate adoption. The role of peer influence in altering physician behavior and reducing overuse is increasingly recognized [16, 32, 33]. Cancer care features rapid diffusion of new technology and treatment modalities, and involves close collaboration among physicians and across specialties where peer influence is particularly relevant. Breast MRI not only affects surgical planning but also other aspects of care (e.g., neoadjuvant chemotherapeutic response and radiation therapy treatment planning) [34, 35]. This likely explains why greater involvement of medical and radiation oncologists in a surgeon’s patient-sharing network was associated with higher adoption of perioperative MRI in our study. Physician network-based behavioral interventions [36] targeting networks with such features may be more effective in reducing overuse of perioperative MRI. Similar future research may help deter the diffusion of other unproven technologies or promote the adoption of guideline-recommended or evidence-based best practices (e.g., via key opinion leaders within networks) [37].

Increased use of perioperative MRI in our study, especially in the high adoption group where MRI use more than doubled its baseline rate of 33.0% to 71.7%, is concerning. Other studies also demonstrated a general increase in use of perioperative breast MRI [1, 21, 38–41]. In a recent survey of breast cancer surgeons, 54.6% believed breast MRI helped their surgical planning and 58.3% anticipated increasing its use over the next 5 years; only 2.3% reported increased mastectomy rate as a drawback of preoperative MRI and just 28.9% reported awareness of practice guidelines [42]. Thus, enhancing provider education may be one area for improvement. Moreover, the American Society of Breast Surgeons released a Choosing Wisely guideline in 2016 recommending against routine use of breast MRI in patients newly diagnosed with breast cancer [6]. Research evaluating more recent trends in perioperative MRI use can inform whether such clinical guidelines may effectively reduce overuse.

Our study also showed that physician networks with slower MRI adoption were able to reduce mastectomy use during a time-period where the national trends in mastectomy use were increasing [43]. Although we cannot make causal inferences, this finding is supported by prior research reporting that perioperative MRI increases patients’ likelihood of undergoing more extensive surgery such as mastectomy [6]. For most women with early stage breast cancer, breast conserving surgery provides at least equivalent clinical outcomes when compared to mastectomy and allows women to preserve the breast [44]. Yet routine use of MRI can lead to overutilization of mastectomy [6]. Therefore, leveraging the role of physician peer influence to promote judicious use of perioperative MRI may have downstream impact on subsequent patient care as well.

Despite an overall concern for overutilization, our finding about differential rate of MRI adoption among physician networks with different patient compositions regarding income and race calls for attention to potential disparity. This finding is consistent with other studies showing lower use of screening MRI among non-White women and women with a high school education or less [45, 46]. Further research is needed to elucidate whether socioeconomically disadvantaged patients are deprived of medically indicated MRI use or whether their differential receipt of perioperative MRI is a marker for inequitable breast cancer care in general.

We recognize several limitations of this study. First, our sample focused on elderly patients with fee-for-service Medicare. Hence the findings may not generalize to younger patients or patients with other types of insurance, where use of perioperative MRI may be even higher [38, 47]. Second, with limited information on provider characteristics, we could not assess other features of physician networks that might influence MRI adoption (e.g., clinical experience and institutional setting). Third, although we accounted for comprehensive tumor characteristics, some relevant factors (e.g., breast density) were not available in our data and might confound the variation in MRI use across physician networks [47]. However, prevalence of dense breasts decreases with age and most women in our sample (age 66–94) would not have dense breasts [48]. Fourth, since we identified physician patient-sharing networks within HRRs, our observed variation in MRI adoption across physician networks may be confounded by practice variation across HRRs. However, in a sensitivity analysis limiting to 97 physician networks in HRRs that had more than one physician network, the different patterns of MRI adoption across the three trajectory groups were very similar to those observed in the primary analysis, supporting the robustness of our findings regardless of number of physician networks per HRR. Prior research also suggests that between-network variation is as important as between-HRR variation in explaining variability in MRI adoption, and between-network variation independently contributes to overall variation in MRI adoption [16]. Fifth, when applying growth mixture modeling, we were unable to account for variability in the uncertainty of estimated rates of perioperative MRI across physician networks (due to different number of patients involved in each physician network and in each time-period). Further methodological advances in this area would enable more rigorous trajectory analysis in future research. Finally, we identified physician networks based on billing relationships documented in claims. Future studies using medical record data to capture other patient-sharing relationships among providers [49, 50] may offer additional insights.

## Conclusions

In summary, over a time-period when perioperative MRI was rapidly adopted into clinical practice nationwide, physician patient-sharing networks differed substantially in their trajectory of adopting perioperative MRI, with some maintaining a low rate of adoption. The distinct adoption trajectories have important implications for subsequent surgical management. Leveraging the impact of physician peer influence may offer a new strategy for addressing overutilization in medical care.

## Supporting information

S1 Table Current Procedural Terminology/Healthcare Common Procedure Coding System codes used to identify perioperative magnetic resonance imaging and breast surgery. (DOCX)
