# Supplementary material for: Perioperative magnetic resonance imaging in breast cancer care: Distinct adoption trajectories among physician patient-sharing networks
Source: PLoS One. 2022 Mar 15;17(3):e0265188. doi: 10.1371/journal.pone.0265188 (PMC8923453; doi:10.1371/journal.pone.0265188)
Supplement: S1 Table — (DOCX) [file pone.0265188.s001.docx]

**S1 Table. Current Procedural Terminology/Healthcare Common Procedure Coding System codes used to identify perioperative magnetic resonance imaging and breast surgery**

|  | **Current Procedural Terminology (CPT) codes** | **Healthcare Common Procedure Coding System (HCPCS) codes** | **International Classification of Diseases, 9th revision (ICD-9), procedure codes** |
| --- | --- | --- | --- |
| **Magnetic Resonance Imaging** | 76093, 76094, 77058, 77059 | C8903, C8904, C8905, C8906, C8907, C8908 |  |
| **Breast Conserving Surgery** | 19110, 19120, 19125, 19126, 19160, 19162, 19301, 19302 |  | 85.20, 85.21, 85.22, 85.23, 85.25 |
| **Mastectomy** | 19180, 19182, 19200, 19220, 19240, 19303, 19304, 19305, 19306, 19307 |  | 85.41, 85.42, 85.43, 85.44, 85.45, 85.46, 85.47, 85.48 |
